# India’s NCD strategy in the SDG era: are there early signs of a paradigm shift?

**DOI:** 10.1186/s12992-018-0357-6

**Published:** 2018-04-25

**Authors:** Shinjini Mondal, Sara Van Belle

**Affiliations:** 1 Department of Family Medicine, McGill University, 5858 Ch de la Côte des Neiges, Montreal, Quebec, H3S 1Z1 Canada; 2 Health Policy Unit, Department of Public Health, Institute of Tropical Medicine, Nationalestraat 155, 2000 Antwerpen, Belgium

**Keywords:** NCD, Intersectoral, Governance, India, SDG

## Abstract

**Background:**

The Sustainable Development Goals (SDGs) are seen in most corners as the embodiment of a more inclusive and holistic development approach, key to addressing the numerous and urgent challenges the world faces. In the health realm, a true SDG approach will require a five-fold paradigm shift according to Buse and Hawkes. This article explores whether early traces of this paradigm shift can already be witnessed in the Indian context, focusing on Non-Communicable Diseases (NCDs) more in particular.

**Discussion:**

By now, NCDs make up a large health burden in India, both individually and on the health system. Inspired by an SDG vision, tackling NCDs will require a comprehensive approach rooted in preventive, curative and rehabilitative services. In India, some early momentum in this respect can already be witnessed, certainly in addressing the first two challenges identified by Buse and Hawkes, leadership and intersectoral coherence, and a shift from treatment to prevention. A central plan addressing health through an inter-sectoral approach has shaped the trajectory so far, moving away from silos to engagement with sectors beyond health. New guidelines addressing comprehensive primary healthcare propose a community outreach and preventive approach for NCDs. At a broader level, NCD prevention is also closely linked to tackling the so called “commercial determinants of health” and will require among others strong (central and state level) regulation, teaming up with global advocacy networks and capitalizing on global frameworks, where they exist. Strong political leadership will be indispensable for this, and is according to Buse and Hawkes closely linked to seeing health as a right and the government as accountable when it comes to providing for the right to health through its policies and actions.

**Conclusion:**

National stewardship will thus be key, via a more adaptive network governance structure with the central level coordinating with the state level to ensure implementation, while also engaging with other stakeholders, sectors, the private sector and civil society. As one can expect, networked governance, necessary for the battle against NCDs, is a work in progress in India. In sum, some of the early (paradigm shift) signs are encouraging, but by and large it is still too early to assess whether a real paradigm shift has taken place.

## Background

Countries around the world have been implementing the Sustainable Development Goals (SDGs) for over a year and a half now. The SDG agenda has been welcomed in many corners for its more holistic and participatory approach to the world’s most pressing issues, as compared to the Millennium Development Goals (MDGs) era. In global health, the SDG framework presents a window of opportunity to open up thinking on global health, break out of mono-disciplinary thinking and vertical paradigms, and unify policies across sectors. However, the framework also presents global health governance challenges, according to Koivusalo, there is a risk of further diluting or fragmenting, the -already scattered- mandate of global health across the UN agencies [1]. It remains to be seen whether the health policy benefits of the SDGs will outweigh the potential drawbacks in the years to come.

In order to address the – in many ways interconnected - SDG goals, multisectoral coordination and more systematic and strategic partnerships with international organisations, local authorities, civil society, business and the private sector will be essential in countries across the globe, to not only implement these transformative goals but also develop effective monitoring and accountability frameworks [2]. Put differently, a fundamentally different set of (global and national) health policy frames and governance models will be required to reach SDG goals and targets by 2030.

In our view, the example of (tackling) NCDs in the Indian context is uniquely suited to explore whether a national policy shift in paradigm is already starting to happen, in line with the abovementioned SDG paradigm, policy & governance changes required for the new era. Due to the multiple determinants of NCDs and the need to focus on preventive, curative and rehabilitative aspects, tackling the NCD-related SDG target 3.4 - “by 2030 reduce by one-third pre-mature mortality from non-communicable diseases through prevention and treatment, and promote mental health and wellbeing” - will require going far beyond the health ministry, not to mention the many global-national NCD pathways which link global policy discourse and trends to national policy implementation. [3] In a way, perhaps even more so than Universal Health Coverage (UHC), NCDs are the quintessential SDG health target, NCDs also have numerous other links with targets under other SDG goals (i.e. other than goal 3, the “health SDG”).

More specifically, we will examine whether in the battle against NCDs in the Indian context, substantial structural changes can already be witnessed, using the five-fold paradigm shift identified by Buse and Hawkes that needs to happen if global and national policy makers are to achieve health goals and targets under the SDG agenda. The outlined five key challenges: leadership and intersectoral policy coherence on structural drivers of health; shifting focus from treatment to prevention, identifying effective means to tackle the commercial determinants of ill health; promoting rights-based approaches and finally, enhancing civic engagement and ensuring accountability [4]. Although Buse & Hawkes do not use the term in their paper, given the complex nature of institutions and partnerships required to achieve SDG health goals (and certainly the multi-pronged NCD challenge), we also argue that a networked governance model, fit for the SDG era, will be needed. ‘Democratic networked’ or interactive governance is increasingly considered as the ‘best fit model’ for national governments to face current complex realities [5]. The network governance model starts from the need to negotiate and seek broader collaboration, building relational contracts over time in the process [6–8] Networks work on models of ‘reciprocity’ and form a complex ‘web of influence’ [9] As is the case in other countries, the NCD challenge in India is a complex one, and can only be addressed by networked governance.

### India’s burden of non-communicable disease and its multiple determinants

At the start of the SDG era, India faces a double disease burden. Concrete measures need to address the rising epidemic of NCDs but also keep the momentum towards progress on preventable infectious diseases, maternal and child mortality. NCDs along with injuries make up the largest disease burden in India (62% of DALYs) [10], jeopardize sustainable growth in India and impact the productivity of individuals. The economic cost for the health system is also increasing, as NCDs contribute 40% of inpatient admissions and 35% of outpatient ones [11]. At the individual level the loss of productive life years due to NCDs in India has been estimated to be one of the highest in the world [12]; this also causes considerable loss of income at household level. Out-of-pocket expenses in case of hospitalisation for NCD treatment come from household savings and income and borrowing money from family and relatives in 45% of the cases [11]. The evidence also suggests that younger people are disproportionately affected [13], and that the NCD burden is rapidly increasing in poorer parts of the population [14], thus posing a challenge to economic growth and cohesion of the country in the medium term.

NCDs have multi-factorial and complex causes. Risk factors can be categorised as modifiable/behavioural risk factors and non-modifiable/individual risk factors. According to WHO, an important way to control NCDs is through reducing the associated behavioural risk factors like smoking, alcohol use, physical inactivity and unhealthy diets [15]. These behavioural risk factors are closely linked with other social determinants like inequitable access to healthcare, poverty, gender, dietary factors and education [16]. The lack of physical exercise and lower intake of fruits and vegetables, coupled with unhealthy food habits is rapidly progressing in urban poor populations [17].

All around the globe, the rise of NCDs is linked to a rapid pace of economic growth and relentless urbanization, accompanied by lifestyle changes which has leading to increased exposure to NCD risk factors [18]. In India, a country characterised by a booming economy and massive urbanization, the rapid transition towards a modern lifestyle for many is obvious. [19]. Urbanisation also led to a greater demand in terms of physical infrastructure and services in urban areas, especially in urban slums where communicable diseases and NCDs co-exist. Unfortunately, access to the formal health sector is poor in slums, with the consequences you can imagine [20, 21]. In response, the Indian Government included the National Urban Health Mission (NUHM) under the National Health Mission (NHM) to cater to the unique challenges of the urban population by improving access to health services and strengthening public health systems. The NUHM also aims for synergies with other urban initiatives of water, sanitation, housing and environmental factors [22].

Globalization also led to more open markets for transnational companies of food, alcohol and tobacco, adding further to the risk factors for NCDs [23]. Tobacco is a well-known NCD adversary, of course, but the close link between dietary consumption of salt, sugar, oils and the risk to develop NCDs is also well established. The Indian economy opened up in the nineties, and the more open market for multinationals has, as expected, contributed to higher levels of exposure to the risk factors, via multiple pathways [24, 25], and a higher NCD burden in India. The impact of globalization processes on NCDs is not all negative, though. Transnational advocacy networks, global regimes and treaties (like the FCTC), also present opportunities in the fight against NCDs [26].

## Discussion

In this section we will describe how India addresses the NCD burden at the start of the SDG era. We use the five abovementioned challenges of Buse & Hawkes **-** leadership and inter-sectoral policy coherence, shifting focus from treatment to prevention, regulating “commercial” determinants promoting poor health conditions, promoting rights-based approaches and a commitment for civic engagement [4] to assess India’s current policy and governance response to reducing the NCD burden. We will also propose how these five shift can be strengthened and better implemented in Indian context.

### Leadership and intersectoral policy coherence

Across the world, addressing NCDs from a more holistic (well-being) angle will require interventions beyond the ministry of health for prevention, cure and rehabilitation. In India, the leadership to steer the country towards the SDGs is found in NITI Aayog, a premier policy think tank of the Government of India (GoI) under the chairmanship of the honourable Prime Minister. The primary function of this organization is to provide strategic design and directives for GoI policies and programmes. It also advises the centre and states by providing technical support.

In 2016, along with the Ministry of Statistics and Programme Implementation (MoSPI), a unique exercise has been initiated to develop indicators that reflect the targets and goals for SDGs [27]. This draft mapping exercise is a vital step, whereby key programmes and sectors addressing the targets have been identified. A key nodal department was also identified for each goal. Furthermore, each target has been assessed by the central scheme or programme that addresses it and has been classified as ‘core of the core’, ‘core’ or ‘optional’ in order of importance. This exercise also identified other concerned ministries and departments that can play an important role in achieving the particular target or goal. For example, for SDG goal 3 the identified nodal ministry is the Ministry of Health and Family Welfare, and core centrally sponsored schemes range from the National Health Mission, Human Resource in Health and Medical Education, National Mission on AYUSH, Integrated Child Development Service to the National AIDS & STD control programme. Among other concerned ministries and departments are: Ministries of tribal affairs, Home Affairs, Women and Child Development, Drinking water and sanitation, Food processing industries, Road Transport and Highway, Environment, forest and climate change. In a clear shift from the MDG era, this comprehensive exercise does spell out very clearly that a siloed approach will not work anymore with the SDGs and a more integrated approach needs to be adopted. Given the range of determinants, this is even more the case for the NCDs challenge (as part of the overall SDG health effort).

To take this forward, NITI Aayog organized a consultation, in February 2016, to engage relevant partners from other ministries, states, international organisations, states and academia [28]. This exercise led to a roadmap for the SDGs. A detailed plan of action and actual implementation with multi-sector co-ordination now comprises the next step forward, the most challenging one obviously.

A few months later, in May 2016, the Ministry of Health and Family Welfare and WHO’s Country Office in India organised a national consultation [29]. At the end of this consultation ‘The Delhi Commitment on sustainable Development Goal for Health’ was released [30], further marking India’s commitment to the achievement of SDG Goal 3. This document acknowledges that health and wellbeing are core pillars for happier societies, economic growth and sustainable development, and require access to services without financial burden. It also highlights that the solution of health issues requires cooperation beyond the health sector and it pushes for providing universal health coverage through strengthening of the existing systems to provide people-centred health services.

Granted, to some extent the importance of a multi-sectoral approach to address the NCD burden in the country was already highlighted in the MDG era, with a key role for the Ministry of Health, as advocated by the national multisectoral action plan and monitoring framework for NCDs [31]. India also adopted WHO’s Global action plan for NCDs 2013–2020, and developed its own national indicators, aiming to reduce premature deaths by 25%. The national programme for prevention and control of cancer, diabetes, cardiovascular disease and stroke (NPCDCS) also stresses on convergence and integration of cross cutting components of intervention for effective implementation [32]. The report of the working group on NCDs for 12th Five Year Plan made a case for providing comprehensive care for NCDs by reducing risk factors, promoting health promotion, prevention and rehabilitative services.; through different levels of healthcare system and engaging sectors beyond health, like Ministries of Environment, Agriculture, Women and Child Development, Human Resource development, Rural Development, Empowerment & Social Justice, and Urban development [33]. However, the (abovementioned) SDG leadership displayed by NITI Aayog and strategy to systematically map and engage sectors beyond health is clearly new, though building on previous initiatives and broadly in line with the approach required in the SDG era.

The ‘networked governance’ needed to address many of the SDGs (and certainly the NCDs) is still a work in progress. For example, more efforts need to be made to involve the private sector and CSOs more systematically (and in a transparent way), with the central government – in the case of NCDs, the Ministry of Health and Family Welfare - as overall “steward”. In order to tackle NCDs decisively, the MoH will need to go beyond its traditional role of priority setting and health policy development and play increasingly the role of a “policy broker”, negotiating pro-actively with other ministries, sectors and stakeholders (including the private sector and CSOs). As India features a range of diverse states with different contexts and NCD realities, a flexible or adaptive network governance structure with steering at national level, while enabling coordination with diverse Indian states, will be a must. Encouragingly, the recent National Health Policy 2017 identified the SDGs as of ‘pivotal importance’ and recognized that alignment of national strategies and global approaches is required [34]. Coordination mechanisms with non-health ministries at national and state levels are also being institutionalized.

All in all, the comprehensive efforts to bring together all sectors and stakeholders and engage in the development of shared responsibilities for NCDs highlight the priority setting and leadership displayed at the central level to move forward and transition from the MDG to the SDG era, ensuring, in the words of Buse and Hawkes, “leadership for intersectoral coherence and coordination on the structural drivers of health”.

### Shifting focus from treatment to prevention through locally-led, politically-smart approaches to a far broader agenda

Buse & Hawkes also emphasize that the focus needs to be “shifted from treatment to prevention, through locally-led, politically-smart approaches to a far broader agenda”. NCDs are a case in point as a number of interventions addressing risk factors have been suggested as ‘best buys’ for NCDs, both in terms of prevention and economic costs associated with the diseases [35]. Involving communities in their healthcare practice and community based services can lead to improved prevention and management. The recently launched guideline (2017), on the screening of NCDs under comprehensive primary care does tries to reach out to larger populations through community health workers and performing of community based screening [36]. This guideline plans to develop an out-reach population based intervention for NCDs, complementing the National Programme for the Prevention and Control of Cancer, Diabetes, Cardiovascular Diseases and Stroke (NPCDCS) [32]. The new guideline builds on the existing community structures and platforms created under The National Health Mission and uses components of Information, Education and Communication (IEC) as an approach for early screening through frontline workers like the Auxilary Nurse Midwife and ASHAs, building community awareness and active mobilization, using already existing community platforms like Gram Sabhas and other community institutions like self-help groups and village health committees [36].

The new guidelines for NCDs under comprehensive primary care is thus encouraging, as they suggest an integrated approach through promotion of early screening, counselling and diagnosis and at the same time try to shift the focus to prevention and promote avenues to raise more NCD awareness and engagement. However, a guideline/policy is often put to the (real) test during its implementation phase. A complex multi-level political setting, (often still lacking) competencies at administrative and technical level, obstruction from vested interests, all play a role in implementation.

### Increasing civic engagement, ensuring accountability

As compared to the “unfinished MDG business”, the NCD epidemic, as Buse and Hawkes note, needs a different approach, focused more on domestically and locally-led initiatives which are globally supported. Put differently, civic and community level-institution engagement (including a potential role in the provision of NCD care), at all levels, will be essential.

At the local level, the trend is certainly encouraging. The recent guideline on NCDs under comprehensive primary healthcare proposes community awareness, active mobilisation and advises states to disseminate NCD- related communication messages at local gatherings, public gatherings and camps to raise awareness at a population level, with support from influential community groups and leaders to gain public participation. The monitoring and accountability for such initiatives can be strengthened by linking them through community based monitoring and planning platforms, established under NHM. These platforms can provide institutional support and ensure that health needs and rights of communities are taken into account. They also provide an opportunity for communities to participate and contribute towards strengthening of health system service delivery.

By way of example, India already has some home based palliative care models of care in Kerala [37]. Initially started as community initiated and community owned platforms, they are now supported through local self-government institutions. The legal framework for these local self-governance structures is provided by the 73rd and 74th constitutional amendment (with mandatory and discretionary provisions for devolutions) [38]. These community spaces have been nurtured through various citizen engagement initiatives. If used in a smart way when implementing the new NCD guidelines, these local level platforms act as societal “control knobs” and accountability mechanisms. At the local level, there is thus certainly potential for another paradigm shift in India – “enhancing civic engagement and ensuring accountability”.

However, on a macro level, ensuring monitoring and accountability will also require investment in developing surveillance systems and identification of reliable indicators. Niti Aayog and MoSPI are currently working on a list of indicators to monitor the progress on NCDs in the SDG context. Baseline measurements and regular assessments and periodic reviews will certainly be needed. The (2017) shadow report by Indian civil society on the SDGs agrees and considers establishing the data monitoring systems as a critical first step. In addition, they emphasize that how data are used to identify priority and investment areas is also critical. To ensure accountability this needs to be a transparent exercise [39]. This SDG involvement by Indian civil society is in line with the transition Buse & Hawkes have transition in mind to cope with the ‘depth’ of the NCD challenge, and health SDGs in general. They emphasize the importance of a vibrant civil society, and civic engagement in all aspects of planning and monitoring.

### Regulating the commercial determinants promoting poor health

Also key in the battle against NCDs, is addressing ‘profitable determinants’ of health, or “commercial determinants of health” as they are often called [40]. Buse & Hawkes argue that “effective means to tackle the commercial determinants of ill-health need to be identified”. That is, as they admit, easier said than done. At the very least, better regulation and negotiation on strict terms with these “commercial determinants” will be essential to prevent the further rise of the NCD epidemic in India and elsewhere [41]. Also in the SDG era, it remains the core duty of the governments to (help) provide basic and essential services for health as well as effectively regulate products and services which have an impact on health one way or another [42]. In the Indian context, this is even more than elsewhere a multi-level governance issue: health remains a state subject under the current governance architecture, while the centre can provide support through centrally sponsored schemes and guidelines. And let’s not forget the global-national pathways (see below).

The challenge the Indian government faces in this respect is partly a capacity issue, as regulatory authorities are often not (yet) sufficiently equipped to do their job well and simultaneously face pressure, lobbying or worse [43]. In addition, many levels and institutions are not (yet) adjusted to the new roles they have to take up in the battle against NCDs. The challenge of NCD-related regulation is even bigger than in other health areas as the government (as a regulatory authority) often faces substantial lobbying or downright obstruction from large transnational corporations.

Given the global nature of the challenge, Keck and Sikkink suggest to engage with ‘transnational advocacy networks’, in order to link and connect actors across states, civil society and international organisations, and facilitate support from regional and international forums, adjusted to the local context. They emphasize that these networks are more than just tools of persuasion: they can also offer political avenues, have the ability to (help) frame issues and expand them beyond (traditional) constituencies, can target and leverage the power of actors and also offer an opportunity to promote accountability politics [26]. For many of the NCD fights ahead, the support of transnational advocacy networks and transnational social movements will certainly be useful, if not essential.

Let’s take the fight against Big Tobacco as an illustration of what is possible at national and other levels in the fight against profit-driven diseases. Some positive and encouraging efforts were already taken to adapt and ratify WHO’s Framework Convention on Tobacco Control (WHO FCTC) in the country [16]. This led to the development of new legislation and implementation already happened at national, sub-national and district levels in this area, even if (a lot) more is needed. Granted, a global legal framework of support comes in handy when “commercial determinants” are involved, as FCTC proves.

As is the case for many other SDG challenges, “effective means to tackle the commercial determinants of ill-health” also require a smart networked governance approach in our opinion, whereby the Ministry of Health and Family Welfare remains the “steward” and sets out the direction on NCDs and other SDG health targets. Suffice to say, a lot of work still lies ahead in this area. Smart networked governance requires, among others, (more) structural engagement with the private sector (both national and multinational corporations), but the often conflicting interests of the business sector also need to be firmly kept in check, via effective national regulatory bodies with sufficient capacity, engaging with transnational advocacy networks like the reinvigorated NCD Alliance, or capitalizing on global governance mechanisms & treaties if they exist (such as the FCTC). Of course, democratic networked governance should also involve a vibrant national civil society, both in the planning & monitoring stage. The stakes are high, as one can gather from the label ‘commercial determinants’. Both Indian and global policy makers will have their work cut out in this respect.

### Promoting a rights-based approach

Finally, Buse and Hawkes also consider a rights-based and social justice approach as essential for well-being and sustainable development, even if they admit that a clear articulation of a rights-based approach in the SDG health goal is – sadly - lacking. In India, the National Health Policy 2017 raised the question whether health care needs a health rights bill as is the case for education. The policy document emphasized the need to move towards the right to health but acknowledged that it is difficult to perceive the right to health without strengthening health systems and providing basic infrastructure and financing. It also pointed out that the right to healthcare requires efforts beyond the health sector and thus a need to work on issues of water, sanitation, nutrition, literacy and poverty [34]. So there are a few (limited) hints on need towards shift to a rights-based approach in India, at the level of certain policy documents at least. However, more will be needed.

Besides, this is not just an Indian gap. At the global level, in addition to the ‘rights’ gap already identified by Buse & Hawkes in the SDG health goal, Gruskin et al. also describe the lack of a human rights approach in the ‘Global Action Plan for the Prevention and Control of NCDs 2013-2020’. They suggest that a real human rights framework provides robust norms and directives, describes legal responsibilities of government and mechanisms to enhance accountability to guide the policy agenda and priority setting for NCD prevention and control [44]. Suffice to say, this will be one of the most challenging paradigm shifts to achieve, not just in India, but all around the globe.

## Conclusion

In this article, we have tried to assess whether signs of the five-fold paradigm shift deemed necessary by Buse & Hawkes to achieve the health SDG targets can already be witnessed in the Indian context, zooming in on the fight against NCDs. By and large, in the early days of the SDG era in India, most progress has been made in terms of “ensuring leadership for intersectoral coherence and coordination on the structural drivers of health”, and “shifting the focus from treatment to prevention through locally-led, politically-smart approaches to a far broader agenda”. “Identifying effective means to tackle the commercial determinants of ill-health” is a tall order in most countries around the world, and India will certainly be no exception, but especially in terms of ‘rights-based approaches’ and ‘enhancing civic engagement and ensuring accountability’, the country still has its work cut out.

In India, the health SDG (including NCD) governance structure is still largely inspired by a “health as an investment” approach, in spite of the ‘rights based’ rhetoric in some policy documents. The health as an investment approach, draws from theory of human capital and suggest that, investing in health has direct and indirect benefits on labour supply and productivity [45]. Health as a right’s based approach, rests to ensure that people live healthy lives and there are robust norms, legal instruments and mechanisms for accountability through government commitment [44]; and has been advocated through Universal Declaration of Human Rights [46]. Though in a recent Lancet article on NCDs [44] borrowing a leaf from HIV battle, Horton argues investment and rights-based approaches should complement each other. Advocating for a multi-pronged global NCD strategy, he provided some ways forward which are certainly also relevant in the Indian context: “*Access to medicines for NCDs should be a decisive matter of human rights. Talk the language of heads of state—investing in the prevention and treatment of NCDs is good for economic growth. Link the case against NCDs to major social movements, such as climate action and the resultant health co-benefits. Build new alliances, especially with the child and adolescent health community*.” [47].

Granted, the lack of clear articulation of a rights-based approach in the health SDGs, and continuing focus on economic growth (albeit inclusive growth) in the overall SDG agenda, which conflicts with sustainable development according to some scholars [48], are not very helpful in that respect, and will continue to present huge challenges for the NCD battle (and the SDG agenda in general), and not just in India. But let’s hope the overall SDG framework can still be adjusted down the road, as implementation progresses and lessons are being learnt.

Recently, the Indian investment discourse was in full display at the recent High-Level Political Forum in New York, when India presented its National Voluntary Review as one of a number of countries [49]. In spite of this discourse, however, India continues to spend far less on health than many other countries across the globe, even with an intended budget increase [50]. This continuing public underinvestment in health has severe consequences in terms of (very much needed) health systems strengthening and delivery of health services, and affects the poorest populations the most as the latter still rely mostly on public sector hospitals and services [51].

Given the importance of the public health sector in India, the Ministry of Health (MoH) needs to steer the NCD battle in India, as the main “steward” in a networked governance structure fit for the twenty-first century, coordinating among others with non-health ministries, CSOs, the for-profit sector and other legitimate stakeholders Although in this governance model the MoH tries to work towards a consensus in the governance model, by bringing in relevant insights and perspectives from all sides, while also ensuring policy coherence as much as possible, conflicts within a network will always be a reality. Interests of stakeholders do not always align with the public interest. A rights based approach should ensure accountability of the network at all times. Boosting community level and civil society participation is essential in that respect, as Buse and Hawkes argue. At the local level, there are some encouraging trends in India, but the overall picture the situation for civil society is more worrying, especially in terms of their sustainability [52]. To ensure the accountability, monitoring, boosting of awareness, mobilization, and inclusive and participatory policy dialogue which Buse & Hawkes say are required for the SDG era, India should thus live up (once more) to its reputation of ‘largest democracy in the world’. For no health challenge this rings perhaps more true than for NCDs: accountability mechanisms based on a rights based approach will require a firm push from civil society (in combination with legal tools, where possible) to make governments at various levels realize and fulfil their duties, and take on the ‘commercial determinants of health’. Evidence from public health has shown that this approach can indeed improve service delivery and enhance equity and inclusiveness with a focus on the neediest [44]. Some current trends raise caution, however [53].

In conclusion, the SDGs offer a window of opportunity to reimagine health as a social goal, including in India, and as a part of a new social contract. Achieving these goals requires structural governance and policy shifts that are firmly rooted in a rights-based approach. This is an uphill task in a complex and heterogeneous country like India, and the NCD path in particular is treacherous as the fight requires taking on powerful “profit-based” actors. Yet, also in the SDG era, governments at all levels continue to have the moral and ethical responsibility for the provision of equitable services and treat human lives with dignity. Final accountability to the public thus rests with them. Bill Gates was spot on when he called India a key battleground in the SDG era, in [52, 54] an interview from 2016. The ‘battleground metaphor’ sounds perhaps nowhere more apt than in the fight against NCDs. However, it’s too early to tell whether (SDG) justice will prevail.

## Abbreviations

CSOs: Civil Society Organisations
FCTC: Framework Convention for Tobacco Control
GoI: Government of India
IEC: Information, Education and Communication
MDGs: Millennium Development Goals
MoH: Ministry of Health
MoSPI: Ministry of Statistics and Programme Implementation
NCD: Non-Communicable Diseases
NHM: National Health Mission
NPCDCS: National Programme for the Prevention and Control of Cancer, Diabetes, Cardiovascular Diseases and Stroke
NUHM: National Urban Health Mission
SDGs: Sustainable Developmental Goals
UHC: Universal Health Coverage
UN: United Nation
WHO: World Health Organisation
